# Multiple breath-hold segmented volumetric modulated arc therapy under real-time fluoroscopic image guidance with implanted fiducial markers: preliminary clinical experience

**DOI:** 10.1259/bjrcr.20160087

**Published:** 2016-10-07

**Authors:** Tsuyoshi Takanaka, Tomoyasu Kumano, Shigeyuki Takamatsu, Tetsuya Minami, Wataru Koda, Toshifumi Gabata, Osamu Matsui, Kimiya Noto, Shinichi Ueda, Yuichi Kurata

**Affiliations:** ^1^Department of Radiation Therapy, Kanazawa University Hospital, Kanazawa, Japan; ^2^Department of Radiology, Kanazawa University Hospital, Kanazawa, Japan; ^3^Department of Radiological Technology, Kanazawa University Hospital, Kanazawa, Japan

## Abstract

A technique for multiple breath-hold segmented volumetric modulated arc therapy (VMAT) has been proposed under real-time fluoroscopic image guidance with implanted fiducial markers. Fiducial markers were embedded as close as possible to a tumour and the patient was asked to breathe in slowly under fluoroscopy. Immediately after the marker positions on the fluoroscopic image moved inside the planned marker contours transferred from a digitally reconstructed radiographic image at each gantry start angle, the patient was asked to hold their breath and a segmented VMAT beam was delivered. During beam delivery, the breath-hold status was continuously monitored by viewing a pointer in a breath monitoring system, Abches (Apex Medical, Tokyo, Japan), with the aid of a video camera installed in the treatment room. As long as the pointer stayed still, the segmented VMAT delivery continued for a preset period of 15–30 s, depending on the breath-hold capability of each patient. As soon as each segmented delivery was completed, the beam interrupt button was pushed; subsequently, the patient was asked to breathe freely. Because the preset breath-hold period was determined in order for each patient to hold their breath without fail, an intermediate beam interrupt due to breath-hold failure during the segmented beam delivery was not observed. This procedure was repeated until all the segmented VMAT beams were delivered. A case of pancreatic cancer is reported here as a preliminary study. The proposed technique may be clinically advantageous for treating tumours that move with respiration, including pancreatic cancer, lung tumour and other abdominal cancers.

## Background

Many techniques were proposed to manage respiratory motion during treatment. Of these, the passive breath-hold technique may be the simplest. However, the tumour position may not be accurately reproduced among the three different periods of planning CT imaging, pre-treatment tumour localization and beam delivery, thus requiring additional treatment margin to compensate for position uncertainty.

To reduce the tumour position uncertainty, a breath-hold monitor, Abches (Apex Medical, Tokyo, Japan), was developed, which had two fulcrums; one was placed on the abdomen and the other on the breast of a patient.^1,2^ A pointer was mechanically connected to the two fulcrums, thus allowing the pointer to move along with the fulcrums during breathing. It was reported that a thoracoabdominal displacement of 1 mm would lead to a pointer rotation of 4.6°.^1^

Spirometry was also used to reduce position uncertainty,^3^ by assuming good correlation between the tumour position and lung volume. However, Eccles et al^4^ reported that although spirometry provided good intrafraction reproducibility of diaphragm position, interfraction reproducibility was less favourable.

Meanwhile, a real-time fluoroscopic image-guided approach was proposed by comparing the diaphragm position between digitally reconstructed radiography (DRR) and real-time kilovolt fluoroscopic images during beam delivery^5^ to assist the clinical staff with manually starting and interrupting the treatment beam during multiple breath-hold treatment. This technique was based on the spatial comparison between the diaphragm contour drawn on the DRR image and the real-time fluoroscopic diaphragm image; therefore, fixed-field beam delivery such as non-coplanar static fields were employed for treating tumours that move with respiration.

To extend the above fluoroscopic technique to volumetric modulated arc therapy (VMAT) for abdominal tumours, a different approach has been proposed here using implanted fiducial markers. A major difference from the previous method is that the diaphragm contour has been replaced with contours of the fiducial markers. This was because real-time visual comparison between the planned diaphragm contour and the real-time fluoroscopic diaphragm image for each gantry angle was impractical during gantry rotation. Meanwhile, it was reported that the dose delivered by segmented VMAT was sufficiently accurate when beam-on time between interrupts was 15 s or greater.^6^ The purpose of this study was to describe the above procedure and demonstrate its clinical advantage for a tumour that moves with respiration.

## Methods

The proposed workflow starts with acquiring planning CT images under deep inspiration breath-hold condition through two markers implanted as close as possible to a tumour but in different craniocaudal positions; subsequently, the CT images were exported to a treatment planning system (Monaco; Elekta AB, Stockholm, Sweden). A single-arc VMAT plan was created, and the plan was exported to a linear accelerator (Synergy; Elekta AB), equipped with a kilovolt fluoroscopic and cone-beam CT (CBCT) imager, X-ray volume imaging (XVI). Because VMAT beam-on time typically exceeds 60 s, multiple breath-holds were required to complete the delivery. In other words, the single-arc VMAT beam was divided into several subarc VMAT beams, each having different gantry start and stop angles. After completing the planning CT imaging, breath-hold training was provided in order to optimize the breath-hold and free-breathing periods for each patient so that each segmented breath-hold VMAT delivery could be successfully completed.

In order to deliver beams while the implanted marker positions coincided with the planned positions, multiple DRR images were created in the Monaco treatment planning system for each gantry start angle of the segmented VMAT delivery and then transferred to the XVI. The fiducial marker positions were contoured on a DRR image window of the XVI display. Subsequently, the marker contours were manually copied onto a fluoroscopic image window of the XVI display using a transparent sheet and a pen, similar to the procedure described by Takamatsu et al.^5^

Prior to the beam delivery, on-board CBCT imaging under free-breathing condition was performed to adjust the position of the patient's couch by matching bone anatomy between the planning CT and the CBCT images.

Under real-time kilovolt fluoroscopic imaging, the treatment staff, using a microphone, asked the patient to breathe in slowly. When the marker positions on the fluoroscopic image moved inside the planned marker contours, the staff asked the patient to hold the breath and a segmented VMAT beam with a photon energy of 10 MV was delivered. During beam delivery, the breath-hold status was continuously monitored by viewing the pointer in the Abches monitoring system with the aid of a video camera installed in the treatment room. As long as the pointer stayed still, the segmented VMAT delivery continued for a preset period of 15–30 s, depending on the breath-hold capability of each patient. As soon as each segmented delivery was completed, the beam interrupt button was pushed; subsequently, a free-breathing period of 10–15 s was provided to prepare for the next breath-hold. Because the preset breath-hold period was determined in order for the patient to hold the breath without fail, an intermediate beam interrupt owing to breath-hold failure during the segmented beam delivery was not observed.

This procedure was repeated until all the segmented VMAT beams were delivered. It was determined that patients unable to hold their breath at least for 15 s would not be suitable candidates for the procedure. A pancreatic cancer patient who could hold their breath for 20 s was selected for this study after written informed consent was obtained.

## Results

**Figure 1.**
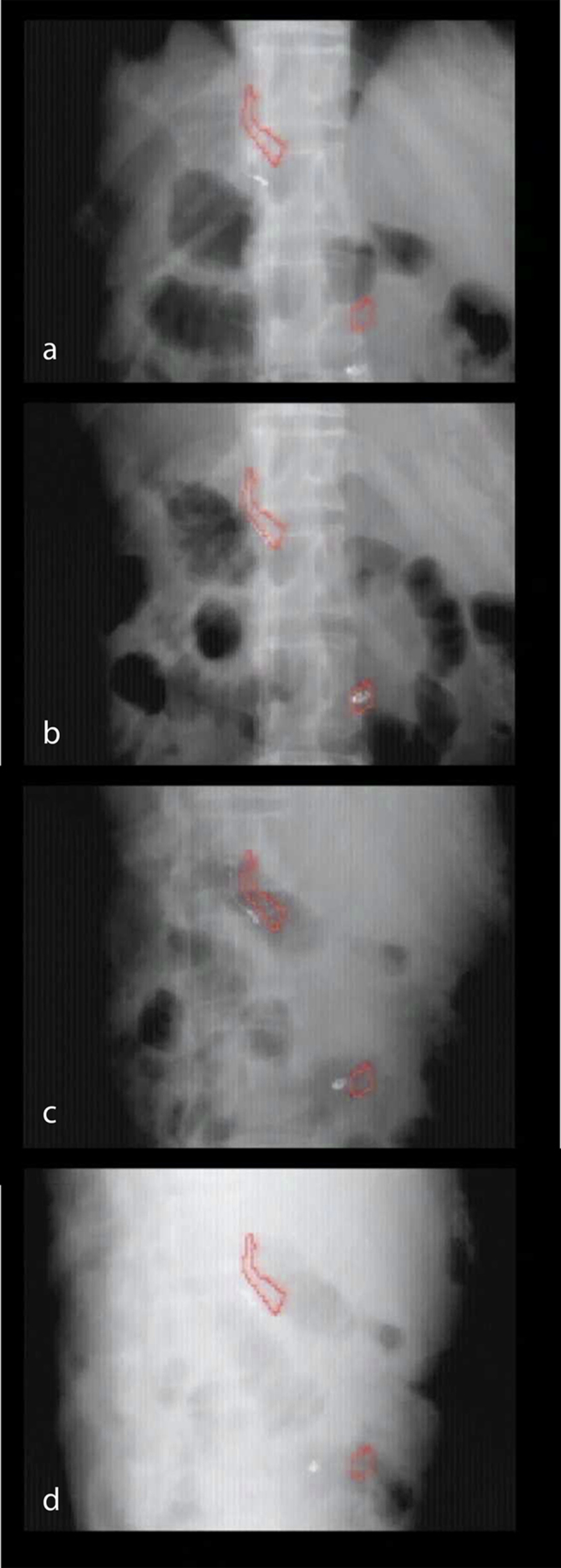
Fluoroscopic images showing the proposed procedure for pancreatic cancer, where two embolization coils were placed in two different peripancreatic arteries. (a) The patient was asked to slowly breathe in at each gantry start angle. The two coil markers on the fluoroscopic image were superiorly approaching the planned marker contours shown in red. (b) When the markers moved inside the planned contour positions, the patient was asked to hold their breath and the volumetric modulated arc therapy beam delivery was started. (c, d) During gantry rotation, the markers moved toward the lateral direction.

Figure 1 shows screenshots of the fluoroscopic images that visually explain the proposed procedure for a patient with pancreatic cancer, where two transarterially implantable platinum embolization coils (Hilal; Cook Medical, Bloomington, IN, USA), were used as markers and placed in two different peripancreatic arteries. (a) Under kilovolt fluoroscopic imaging at a gantry start angle, a patient was asked to slowly breathe in. The markers were superiorly approaching the planned contour positions shown in red. (b) When the markers moved inside the planned contour positions after travelling approximately 5 cm, the patient was asked to hold their breath and the delivery of a segmented VMAT beam was started. (c, d) During the breath-hold period, the markers moved toward the lateral direction while the gantry was rotating. Because the visibility of the markers varied depending on the gantry angle as shown in Figure 1c,d, breath-hold status was more effectively monitored by viewing the pointer position of the Abches monitoring system.

## Discussion

In the proposed procedure, the tumour position was visually monitored by either the implanted fiducial markers at each gantry start angle or the pointer of Abches system during gantry rotation. In the meantime, it was reported that respiratory-gated VMAT resulted in four times longer treatment time when the breathing frequency was 15 cycles min^–1^ compared to non-gated VMAT delivery.^7^ The proposed segmented breath-hold technique may be much faster if the number of beam interrupts is relatively small. In this patient with pancreatic cancer, the total VMAT delivery time for a prescribed dose of 2 Gy per fraction was approximately 3 min with five beam interrupts. The shorter delivery time may ensure more comfort to patients and thus better intrafraction position stability. The marker position differences between the DRR and the kilovolt fluoroscopic images were measured on frontal and lateral views for all the fractions, leading to mean distances of 3.8, 3.9 and 2.9 mm in lateral, craniocaudal and ventrodorsal directions, respectively.

In conclusion, a new multiple breath-hold segmented VMAT technique has been proposed under real-time fluoroscopic image guidance with implanted fiducial markers. The proposed technique may be clinically advantageous for treating tumours that move with respiration, including pancreatic cancers, lung tumours and other abdominal cancers.

## Learning points

1. A multiple breath-hold segmented VMAT technique was proposed using implanted fiducial markers.
2. Real time fluoroscopy was employed to confirm the absolute positions of the markers at each gantry start angle, whilst the Abches pointer was used to confirm the marker immobilization during each VMAT delivery.

## Consent

Informed consent was obtained and held on record.
